# A case report and review of calcinosis cutis

**DOI:** 10.1093/jscr/rjae068

**Published:** 2024-02-13

**Authors:** Tiffany Bender, Michael Burt

**Affiliations:** University of South Dakota Sanford School of Medicine, 1400 W 22nd Street, Sioux Falls, SD 57105, United States; Department of General Surgery, University of South Dakota Sanford School of Medicine, Sioux Falls, SD 57104, United States

**Keywords:** calcinosis cutis, systemic sclerosis, renal failure, soft tissue calcification

## Abstract

Commonly associated with autoimmune and renal disorders, calcinosis cutis is a disorder of systemic calcium deposition in soft tissues. The pathophysiology of such deposition varies based on subtype, therefore treatment options vary not only in terms of severity of disease but also with subtype. This case report describes a 52-year-old female with systemic sclerosis and an extensive past medical history who initially presented with complaints of worsening left lower leg pain, a negative workup for deep vein thrombosis, and an extensive palpable mass in the posterior thigh with erythema, drainage, and purulence. With multiple treatment options exhausted from her autoimmune disorders, she ultimately required surgical resection for her refractory infected calcinosis cutis. Identification of calcinosis cutis subtype in conjunction with appropriate history and physical is crucial to determining indications for treatment.

## Introduction

Calcinosis cutis is a systemic deposition of insoluble calcium throughout skin and soft tissue. Deposition of calcium phosphate crystals accumulates within soft tissue creating subcutaneous nodules. Most nodules (65–83%) are found in the hands. In addition to location, the nodules can also vary in size, ranging from millimeters to centimeters [1]. Calcinosis is categorized into five subtypes: idiopathic, iatrogenic, calciphylaxis, metastatic, and the most common form, dystrophic [1]. Dystrophic forms are most often associated with renal failure and autoimmune disorders, particularly systemic sclerosis (SS). Dystrophic calcinosis cutis is differentiated from other subtypes with normal serum calcium and phosphorous levels [1]. Among patients with SS, it is estimated that 18–49% will develop calcinosis cutis [2]. Longer durations of SS increase a patient’s risk for developing calcinosis cutis. Furthermore, men are more prone to systemic progression [1].

Calcinosis cutis is often initially detected through physical examination and diagnosed clinically but should always be confirmed via imaging or laboratory studies such as serum calcium or phosphorus levels [3]. Multiple modalities can be utilized to confirm the diagnosis, depending on the location of the nodule(s). Ultrasound is the most sensitive form of imaging; however, CT and MRI provide higher resolution images [1]. While a benign disorder, depositions of calcium may accumulate and often result in pain, reduced mobility, ulcerations, infections, and disfigurement [3, 4].

This report describes a 52-year-old female with SS who developed severe calcinosis cutis refractory to numerous treatments and ultimately required surgical management.

## Case report

A 52-year-old female presented to a rural emergency department with complaints of worsening left lower leg pain over 5 days. Bilateral dorsalis pedis and left posterior tibial pulses were detectable with Doppler. A D-Dimer and troponin level were ordered, both of which were elevated. A CBC revealed anemia. Procalcitonin and calcium were within normal limits. Due to concerns for an arterial occlusion or a deep vein thrombosis (DVT), the patient was started on a heparin drip and transferred for further workup.

**Figure 1:**
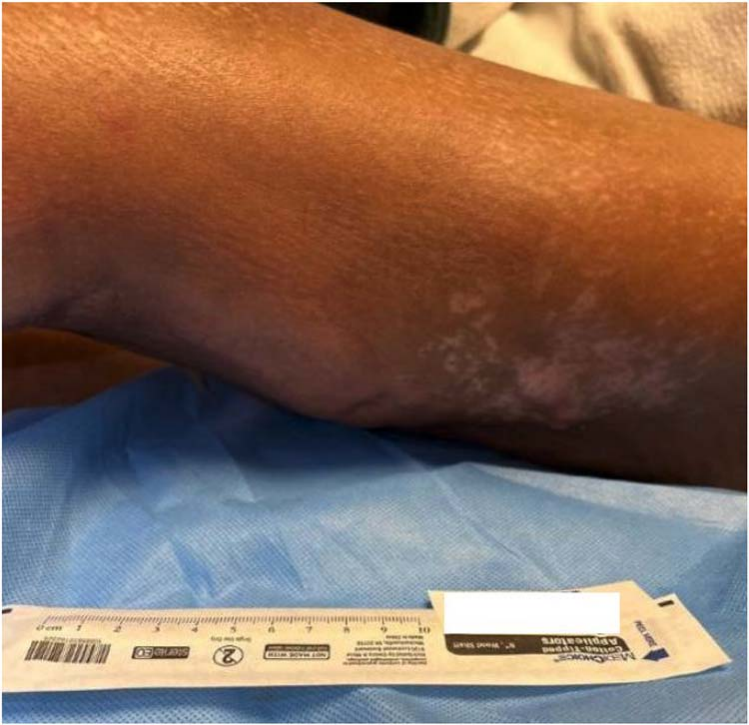
The posterolateral thigh at admission to our tertiary facility.

Upon arriving at our tertiary medical center the patient’s pain persisted despite IV heparin. Her medical history included SS treated with rituximab, type 2 diabetes, rheumatoid arthritis treated with methotrexate, polymyositis, Raynaud’s disease, and lupus. Her lupus had previously been treated with chronic mycophenolate immunosuppression, however, it was held at admission due to infection. Physical exam revealed a hemodynamically stable patient with left lower extremity erythema and warmth to the posterior lateral aspect. The area had scant serous drainage and subcutaneous crepitus upon palpation (Fig. 1). A radiograph of the left knee showed course, nonspecific soft tissue calcifications, but no evidence of acute fracture, dislocation, or osteomyelitis. Arterial duplex and extremity venous ultrasound were negative for DVT or arterial occlusion.

She was admitted for sepsis secondary to cellulitis, started on prophylactic IV vancomycin, and received infectious disease consultation. General surgery was consulted as the area of concern had begun producing copious amounts of malodorous and purulent discharge. Antibiotic coverage was broadened to ceftaroline. Rheumatology assessed the patient and noted she had a particularly aggressive, progressing, and immunosuppression-resistant diffuse SS. Further evidence of the severity of her SS became apparent as diffuse skin thickening and subcutaneous calcifications precluded procedures such as peripherally inserted central catheter (PICC) line placement.

**Figure 2:**
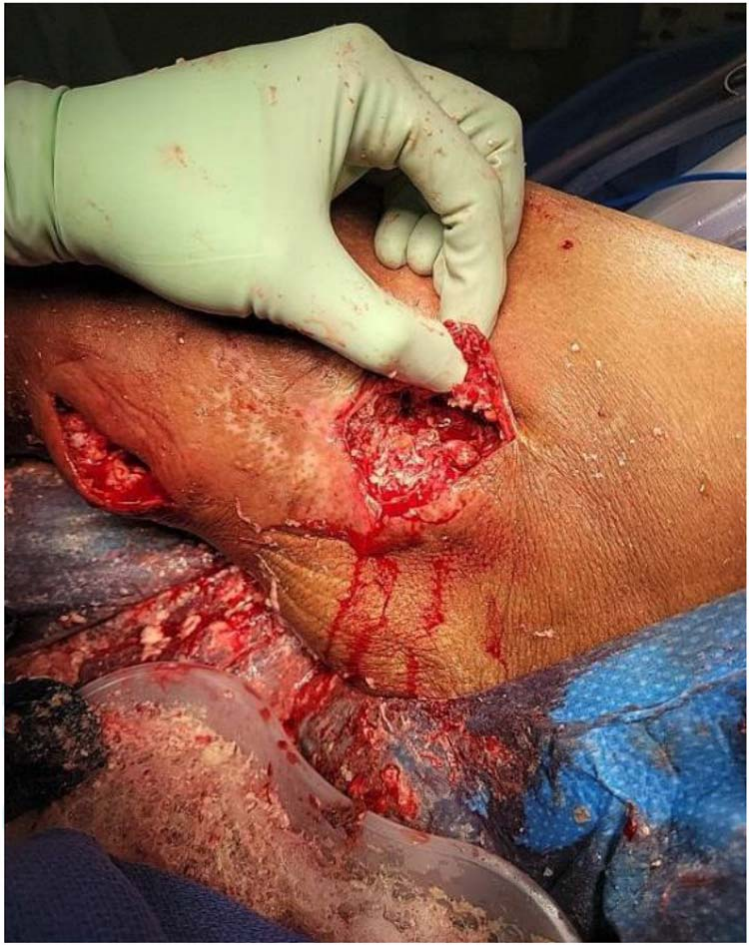
Initial incision, drainage, and debridement of the wound showing extensive coral-like calcifications in conjunction with thick milky non-odorous fluid.

The general surgery consultation revealed the wound had been subtly progressing for ~3 years and had only recently begun to drain fluid. On exam, the left lower extremity was indurated and erythematous with areas of fluctuance and purulent drainage. Therefore, the patient was taken to the OR for incision, drainage, and debridement. Intraoperatively, extensive coral-like calcifications in conjunction with thick milky non-odorous fluid were noted (Fig. 2). Numerous calcification fragments were removed, the wound was cultured, and the area was irrigated. The wound was packed with gauze, wrapped with Kerlix, and received postoperative daily wet-to-dry dressing changes.

**Figure 3:**
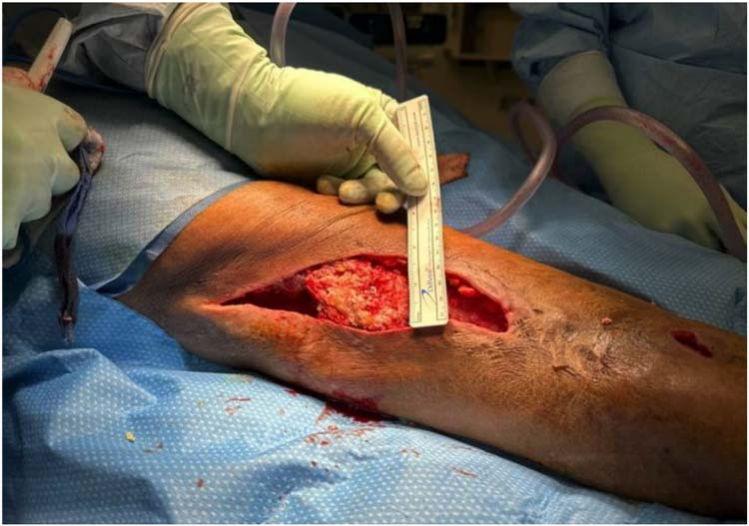
The remaining intact calcified mass before excision.

**Figure 4:**
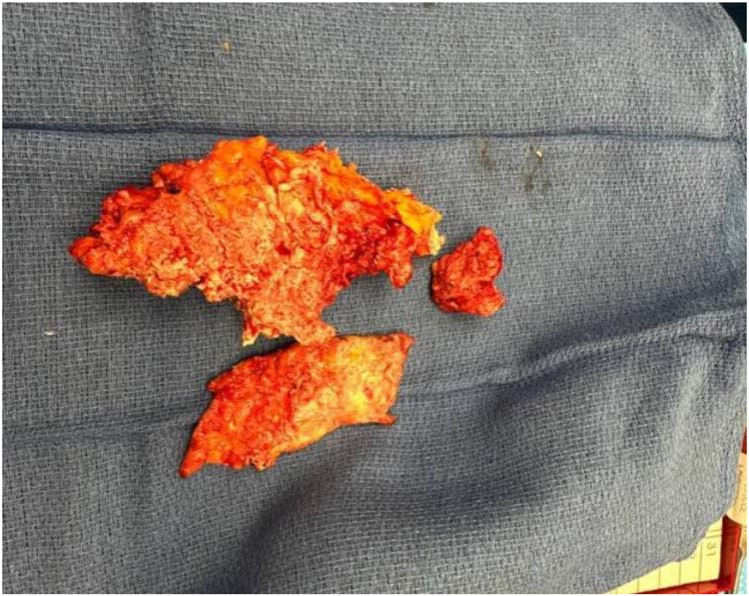
The excised calcified tissue measuring 17 × 8 × 4 cm.

Wound cultures revealed methicillin-resistant *staphylococcus aureus* (MRSA) and *staphylococcus epidermis*; thus, ceftaroline was changed back to vancomycin. Eight days after her initial I&D, she was taken back to the OR for additional debridement. Extensive massive calcified tissue was still evident, measuring 17 × 8 × 4 cm (Fig. 3), and was debrided (Fig. 4) with wound vac placement. The patient returned to the OR on postoperative Days 3 and 16 for wound vac replacement and wound closure with Provena wound vac placement, respectively. Two days later, the patient was stable for outpatient follow-up and was discharged to inpatient rehab.

## Discussion

To date, there is no standardized approach to treating calcinosis cutis. Pharmacologic management ranges from antibiotics, immunosuppressants, bisphosphonates, and colchicine to intravenous immunoglobulins and other biologics [1]. Non-pharmacologic treatment options may include shock therapy, CO_2_ lasers, or surgical excision. Due to this patient’s extensive medical history, many of the aforementioned pharmacologic treatments had been utilized for other comorbidities and proved refractory to the nodules, including methotrexate, colchicine, and numerous antibiotic courses. Furthermore, pharmacologic options were limited due her poor renal function.

Compared with pharmacologic modalities, surgical excision has shown the greatest efficacy with 79–96% improvement [2]. However, surgical intervention is often reserved for treatment-resistant cases due to the risk of complications, poor wound healing, and potential need for skin grafts [1, 4]. In a small retrospective study (N = 11) at Mayo Clinic, all of the patients who received surgical resection experienced positive outcomes compared with variable outcomes with pharmaceutical treatment. Surgery recipients reported 72% complete resolution of nodules with the remaining 27% having partial resolution [5].

## Conclusion

To our knowledge, this is the first report of a patient with aggressive calcinosis cutis of this size and nature. The dimension of this nodule threatened neurovascular structures of the popliteal fossa and the posterior portion of the thigh while also serving as a nidus for infection. Due to the dystrophic subtype, there was no direct target for pharmacologic treatment of a particular mechanism of the pathology, unlike calciphylaxis or metastatic subtypes. The patient’s complex medical history also posed many contraindications to pharmacologic treatment, ultimately making her a candidate for surgical resection.
